# Supplementary material for: Synergistic action of mucoactive drugs and phages against Pseudomonas aeruginosa and Klebsiella pneumoniae
Source: Microbiol Spectr. 2025 Feb 6;13(3):e01601-24. doi: 10.1128/spectrum.01601-24 (PMC11878038; doi:10.1128/spectrum.01601-24)
Supplement: Supplemental Material — Figures S1 to S3; Tables S1 and S2. [file spectrum.01601-24-s0002.docx]

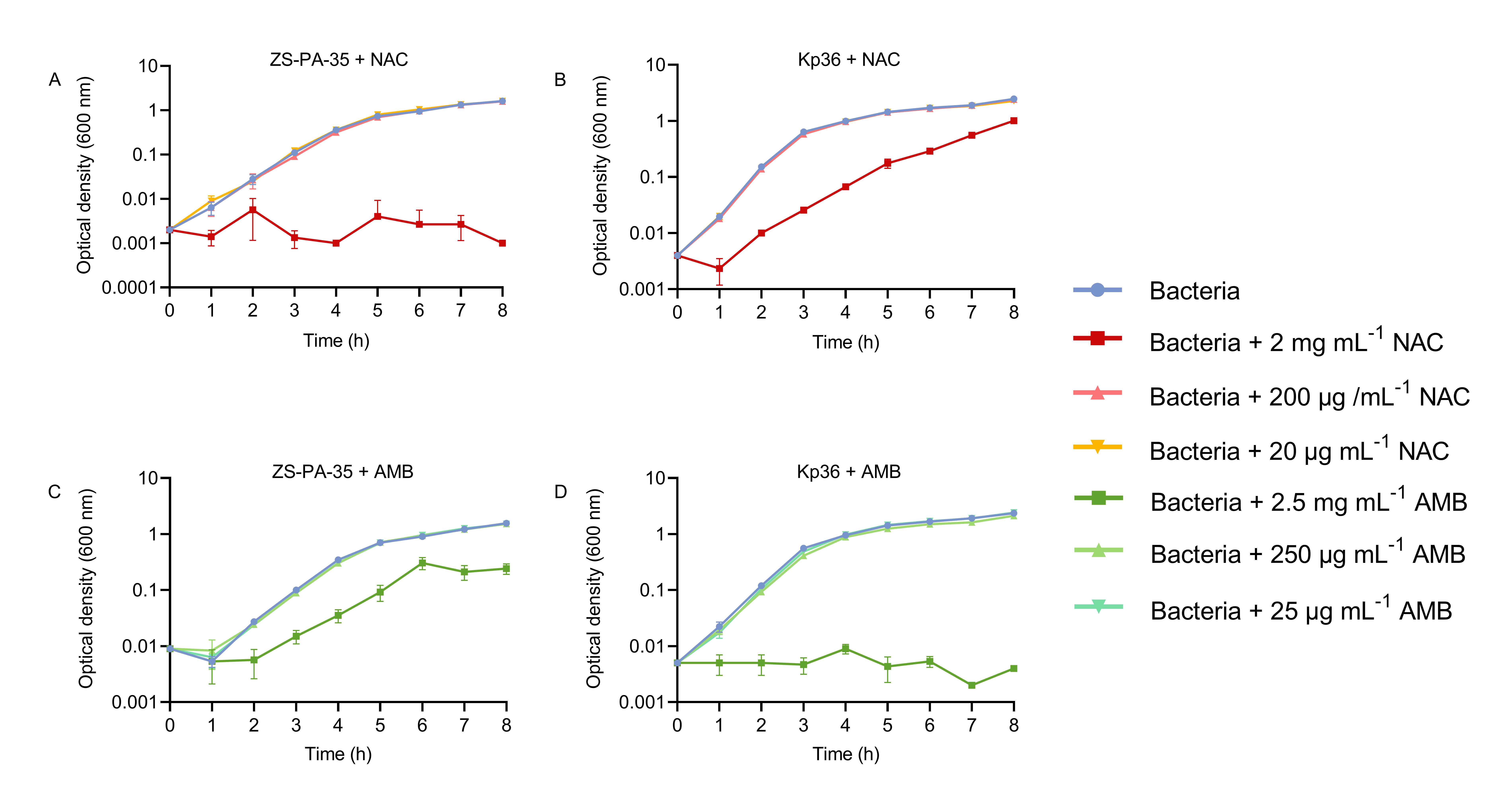
 Fig. S1. Effects of varying concentrations of NAC and AMB on bacterial growth in LB medium. Panels (A) and (C) illustrate the impact of NAC on ZS-PA-35, while panels (B) and (D) display the effects of AMB on Kp36. Experiments were performed in triplicate, with error bars representing the mean ± standard deviation (SD)


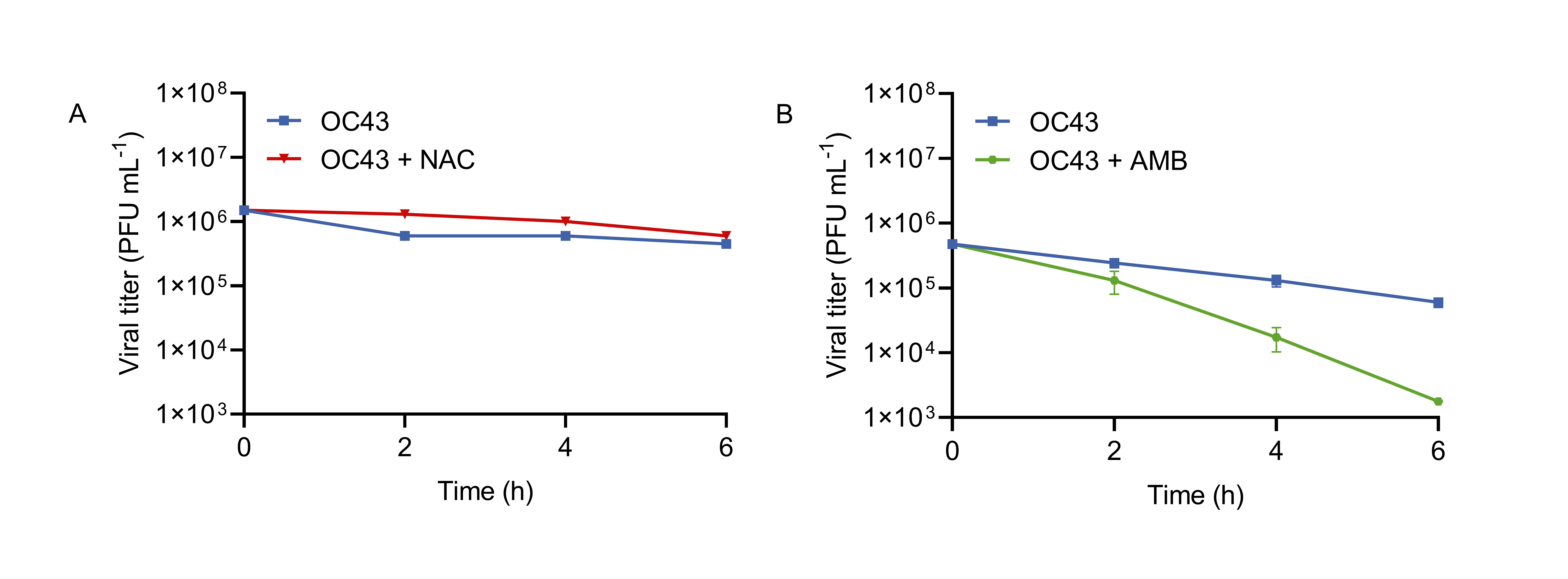


Fig. S2. Effect of NAC and AMB on respiratory coronavirus OC43 in BHK-21 fibroblast cells over 6 h. Panel (A) shows that NAC did not influence the stability of OC43, while panel (B) illustrates a strong antiviral effect of AMB. Error bars in panel (B) represent the mean ± standard deviation (SD).


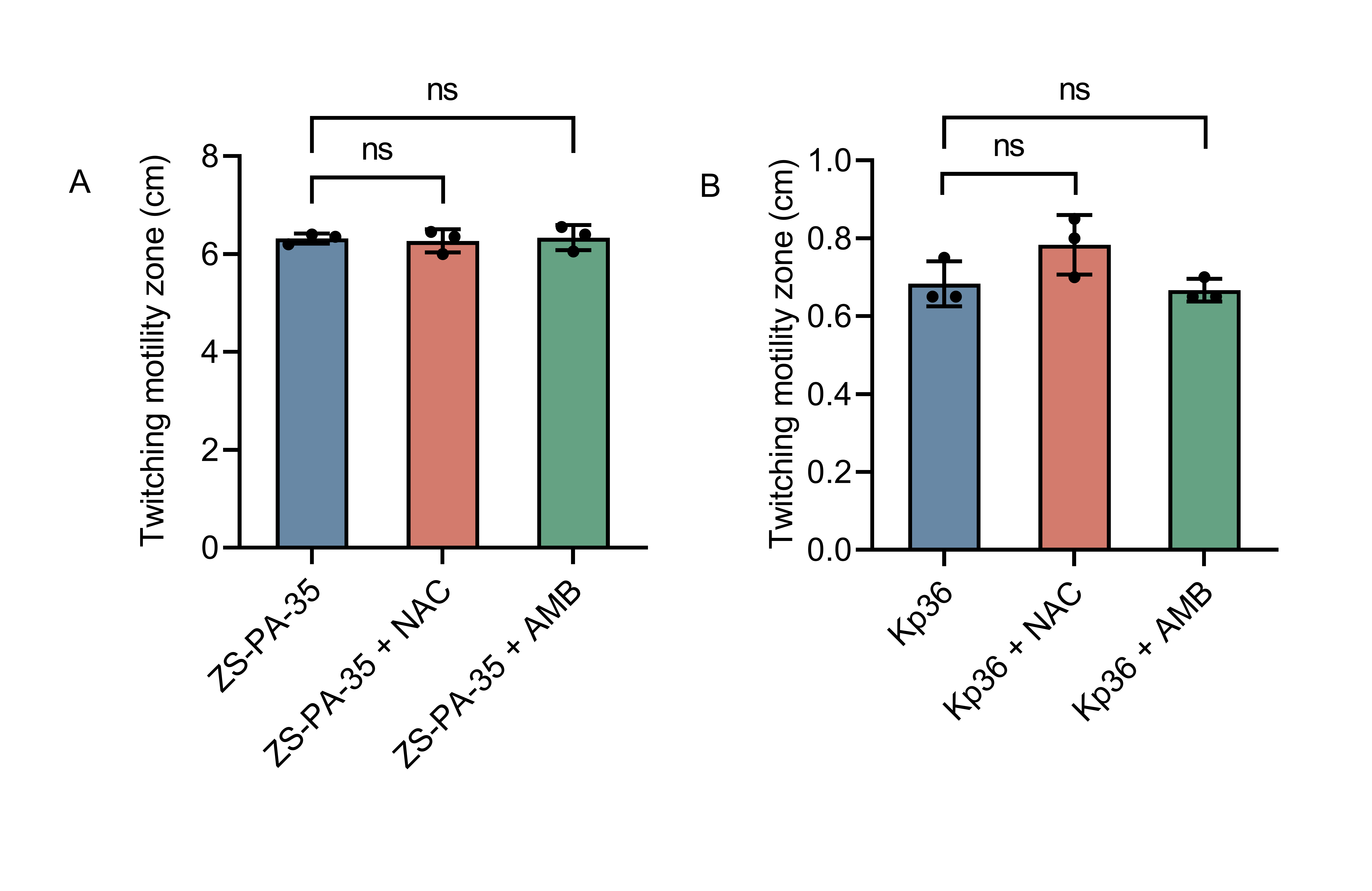


Fig. S3. Effect of NAC and AMB on the twitching motility of ZS-PA-35 (A) and Kp36 (B). Bacterial strains were inoculated at the center of 1.5% soft agar plates containing either NAC or AMB, with control plates lacking these mucoactive agents. Plates were incubated at 37°C for 72 h. After removing the agar, static staining with 0.4% crystal violet was performed, and twitching motility was quantified by measuring the migration distance at the agar-Petri dish interface (cm). Experiments were conducted in triplicate, with error bars representing the mean ± standard deviation (SD).

Table S1 Bacterial strains and phages

| Name | Genotype or relevant markers |
| --- | --- |
| ZS-PA-35 | Wild-type strain, *Pseudomonas aeruginosa*, host for phage phipa2 and phage phipa10 |
| *ΔpilT* | **A ZS-PA-35 mutant with a deletion in the *pilT* gene was resistant to phage phipa2 but remained susceptible to phage phipa10** |
| *ΔgalU* | **A ZS-PA-35 mutant with a deletion in the *galU* gene was resistant to phage phipa10 but remained susceptible to phage phipa2** |
| Kp36 | Wild-type strain, *Klebsiella pneumoniae*, host for phage 117 |
| Phage phipa2 | *Podoviridae*, a 43 kb lytic phage, uses type IV pili as its receptor |
| Phage phipa10 | *Myoviridae*, a 92 kb lytic phage, use O-antigen as its receptor |
| Phage 117 | *Podoviridae*, a 41 kb lytic phage, use colanic acid as its receptor |

Table S2 qPCR oligonucleotides, products, and sources used in the experiment

| Gene | Primer Sequence F/R (5’–3’) | Product (bp) | Bacterial |
| --- | --- | --- | --- |
| *galU* | CTCGATCAGGTCGAAGATGTC  TGAACACCATGGTCGAGAAG | 101 | ZS-PA-35 |
| *pilT* | ATGCTCGACTACCTGAACAAC  ACCAGGCACTTCTTCGATTC | 92 | ZS-PA-35 |
| *rpoS* | CGGAGTTTGACCACGATGAT  GAGAAGGAAGTGGTGGCTTT | 118 | ZS-PA-35 |
| *wcaJ* | GATGGAGAGGTGAAACGGATAC  CCAACCAAATACTCCACCCA | 90 | Kp36 |
| *rpoD* | CCTGAATACCCATGTCGTTGA  GAGCAAGGCTATCTGACCTATG | 112 | Kp36 |

Supplemental File 1: Data sources for statistical analyses***
